# Plasma Androgen Receptor in Prostate Cancer

**DOI:** 10.3390/cancers11111719

**Published:** 2019-11-04

**Authors:** Vincenza Conteduca, Giorgia Gurioli, Nicole Brighi, Cristian Lolli, Giuseppe Schepisi, Chiara Casadei, Salvatore Luca Burgio, Stefania Gargiulo, Giorgia Ravaglia, Lorena Rossi, Amelia Altavilla, Alberto Farolfi, Cecilia Menna, Sarah Pia Colangione, Mario Pulvirenti, Antonino Romeo, Ugo De Giorgi

**Affiliations:** 1 Department of Medical Oncology, Istituto Scientifico Romagnolo per lo Studio e la Cura dei Tumori (IRST) IRCCS, 47014 Meldola, Italy; nicole.brighi@irst.emr.it (N.B.); cristian.lolli@irst.emr.it (C.L.); giuseppe.schepisi@irst.emr.it (G.S.); chiara.casadei@irst.emr.it (C.C.); salvatore.burgio@irst.emr.it (S.L.B.); lorena.rossi@irst.emr.it (L.R.); amelia.altavilla@irst.emr.it (A.A.); alberto.farolfi@irst.emr.it (A.F.); cecilia.menna@irst.emr.it (C.M.); ugo.degiorgi@irst.emr.it (U.D.G.); 2 Biosciences Laboratory, Istituto Scientifico Romagnolo per lo Studio e la Cura dei Tumori (IRST) IRCCS, 47014 Meldola, Italy; giorgia.gurioli@irst.emr.it (G.G.); stefania.gargiulo.94@gmail.com (S.G.); 3 Unit of Biostatistics and Clinical Trials, Istituto Scientifico Romagnolo per lo Studio e la Cura dei Tumori (IRST) IRCCS, 47014 Meldola, Italy; giorgia.ravaglia@irst.emr.it; 4 Radiotherapy Unit, Istituto Scientifico Romagnolo per lo Studio e la Cura dei Tumori (IRST) IRCCS, 47014 Meldola, Italy; sarah.colangione@irst.emr.it (S.P.C.); antonino.romeo@irst.emr.it (A.R.); 5 Department of Urology, Morgagni Pierantoni Hospital, 47121 Forli, Italy; mario.pulvirenti@auslromagna.it

**Keywords:** androgen receptor, prostate cancer, plasma DNA, biomarkers

## Abstract

The therapeutic landscape of prostate cancer has expanded rapidly over the past 10 years, and there is now an even greater need to understand the biological mechanisms of resistance and to develop noninvasive biomarkers to guide treatment. The androgen receptor (AR) is known to be involved in the pathogenesis and progression of prostate cancer. Recently, highly sensitive next-generation sequencing and PCR-based methods for analyzing androgen receptor gene (AR) copy numbers (CN) and mutations in plasma were established in cell-free DNA (cfDNA) of patients with castration-resistant prostate cancer (CRPC) treated with different drugs. The study of cfDNA holds great promise for improving treatment in CRPC, especially in the advanced stage of the disease. Recent findings showed the significant association of plasma *AR* aberrations with clinical outcome in CRPC patients treated with AR-directed therapies, whereas no association was observed in patients treated with taxanes. This suggests the potential for using plasma *AR* as a biomarker for selecting treatment, i.e., hormone therapy or chemotherapy, and the possibility of modulating taxane dose. In recent years, plasma *AR* status has also been investigated in association with novel agents, such as ^177^Lu-PSMA radioligand therapy and PARP inhibitors. This review will focus on *AR* testing in plasma that may have clinical utility for treatment selection in advanced prostate cancer.

## 1. Introduction

Prostate cancer is the most frequent cancer in men and the second most common cause of cancer-related death in Western countries [1]. The disease shows wide clinical variability and molecular heterogeneity. The androgen receptor (AR), a nuclear hormone receptor which, upon activation by androgens, translocates into the nucleus and binds to specific regulatory regions, plays a key role in the normal development of the prostate as well as in the pathogenesis of prostate cancer. The majority of androgen-independent or hormone refractory prostate cancers express AR, thus making it an important therapeutic target in all tumor stages ranging from early to the more advanced disease [2].

The majority of hormone-naïve prostate cancer (HNPC) patients characterized by non-castrate testosterone levels who undergo androgen deprivation treatment (ADT) may become castration-resistant (CRPC). CRPC is defined as radiographic progression and/or increase in serum prostate-specific antigen (PSA) levels despite a low testosterone concentration (<50 ng/mL) [3,4] Despite therapeutic advances in the last two decades, these patients still show a low median survival ranging from approximately 18 to 36 months [5].

**Figure 1:**
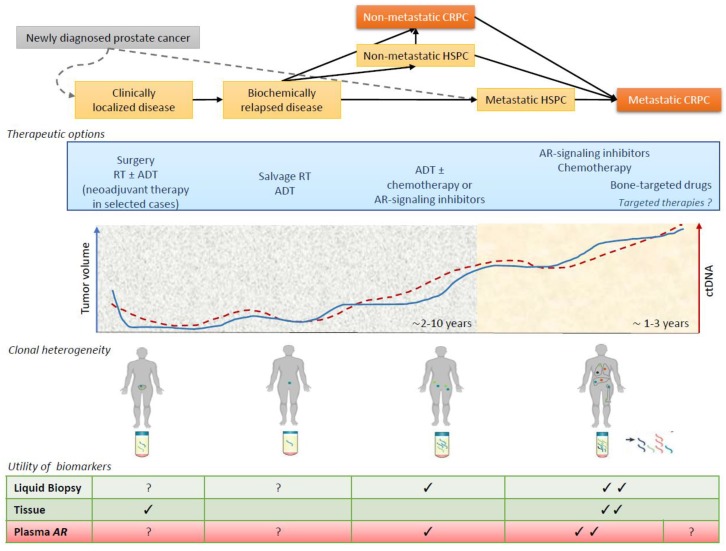
The role of biomarker tools and plasma *AR* status in different clinical states of prostate cancer. A clinical states framework for clinical practice, clinical research, and biomarker tools in prostate cancer with a graphical representation of tumor burden, plasma DNA levels, clonal heterogeneity, and utility of plasma *AR* status over time and in response to therapy. Abbreviations: ADT, androgen deprivation therapy; AR, androgen receptor; CRPC, castration-resistant prostate cancer; ctDNA, circulating tumor DNA; HSPC, hormone-sensitive prostate cancer; RT, radiotherapy.

There are several therapeutic options available for prostate cancer, including radiotherapy, ADT, second generation AR-signaling inhibitors, chemotherapy, and bone-targeted agents. Numerous trials are also ongoing for the approval of novel targeted drugs (Figure 1).

In this paper, we review the literature on the role of AR detected in the plasma of patients with prostate cancer as a novel prognostic and predictive biomarker in different settings of prostate cancer.

## 2. Clinical Prognostic Factors in Prostate Cancer

Currently, prostate cancer treatment decisions (curative treatments or active surveillance) in the setting of localized disease are still based almost exclusively on histological architecture (Gleason score) [6], prostate-specific antigen (PSA) levels [6] and local disease state (TNM) [7], with little attention paid to molecular features, except for some genomic tests using tissue biopsy (Prolaris, OncotypeDx, and Decipher) available for men with localized prostate cancer [8,9,10] and there is early metastasis prediction model based on genomic expression in the primary tumor which could be helpful for identification of aggressive prostate cancer [11]. Prolaris is a panel of cell cycle progression genes that can be measured in both untreated and previously treated patients [8]. OncotypeDx predicts the risk of disease recurrence at radical prostatectomy for men with low-to-intermediate risk prostate cancer [9]. Decipher provides information on genome-wide RNA expression for newly diagnosed patients which can be used to predict the risk of metastasis in men with adverse pathology at radical prostatectomy. Additionally, these assays could help to imrpove prognostic risk stratification to aid treatment decisions [10]. However, they should be prospectively validated, especially with respect to their clinical utility and impact on the cost of localized prostate cancer care.

During disease evolution, further clinical factors are used for prognostication and risk stratification. In patients diagnosed with nonmetastatic disease, baseline PSA levels, PSA velocity, and PSA doubling time are associated with clinical outcome and can be used to select patients whose follow-up requires imaging studies [12].

Although there is no universally accepted prognostic classification for patients with metastatic HNPC (mHNPC), a recent study [13] identified time of metastatic presentation and disease volume as prognostic variables for mHNPC patients treated with ADT. In the last few years, the therapeutic landscape for mHNPC has substantially changed, with the addition of docetaxel or abiraterone acetate to ADT in selected cases based on tumor (number and site of metastasis) and clinical patient characteristics [14,15,16,17]. However, predictive biomarkers are also warranted to facilitate treatment decision-making (single or combination) in HNPC patients.

Several prognostic nomograms, especially in CRPC, have been developed to predict overall survival (OS) and to assess risk stratification in clinical trials [5,18,19,20]. Following a number of recent trials focusing on the treatment of mHNPC [14,15,16] and the introduction of abiraterone or enzalutamide as first-line treatment in most mCRPC patients based on the results of COU–AA–302 and PREVAIL studies [21,22], new nomograms are currently being developed in this earlier setting.

One important aspect to consider in the evaluation of treatment outcome is the biological heterogeneity of prostate malignancy. Thus, disease stratification based on molecular signatures is warranted in prostate cancer, as it has also been shown to aid the prognostic evaluation and management of other epithelial cancers such as breast cancer [23].

## 3. Molecular Factors in Driving Castration-Resistant Prostate Cancer (CRPC) Emergence

Over the years, many studies have consistently shown that the majority of CRPCs are still dependent on AR signaling, which may also play a role in the transition from early to advanced disease, even at low concentrations of androgens. One of the most important objectives of recent molecular studies has been to identify synergistic factors which, together with AR, are involved in prostate cancer progression.

The phase III CALGB 90203 trial [24] aimed to explore the molecular alterations associated with chemotherapy/hormone therapy in the neoadjuvant setting. Targeted DNA sequencing (n = 72 genes) and expression profiling using NanoString platform (n = 163 genes) were performed on prostatectomy-derived tissue specimens of 52 patients enrolled in the study. The most frequent alterations were *TMPRSS2–ERG* fusion (61.5%), *TP53* mutation or deletion (21.1%), *PTEN* deletion (11.5%), *FOXA1* (11.5%), and *SPOP* (7.7%) mutation, with no significant enrichment in post-treated specimens.

In this study [24], no *AR* aberration was observed in the neoadjuvant setting, probably because *AR* amplification and mutations are typically later events in the process of prostate cancer progression (present in 60% of metastatic disease) [25,26]. However, some studies [27,28,29] have shown that AR signaling may also be relevant at early time-points and potentially drive response and resistance to therapy, probably through mechanisms different to those of later AR reactivation in CRPC. Further studies are warranted in this setting (Figure 1). Beltran et al. [24] observed an upregulation of both AR and a specific variant of AR (AR-V7) in the group treated with neoadjuvant therapy comprising docetaxel and ADT. The ratio of AR-V7 to wild-type AR was also comparable in the untreated cases. These findings support the hypothesis that the activity of AR and/or alternative transcriptional enhancers may persist, providing the basis for subsequent castration resistance.

Lastly, genomic and transcriptomic analysis of CALGB 90203 trial [24] demonstrated the upregulation of both neuroendocrine and plasticity genes (such as *RB1*, *T*P53, and *MYC*) [30,31,32], and the downregulation of AR target genes including the *TMPRSS2–ERG* fusion transcript in post-treatment tissue specimens. Thus, neoadjuvant treatment may shape the clonal architecture of prostate cancer with involvement of lineage plasticity and neuroendocrine differentiation, not only in mCRPC but also in early disease, leading to an early adaptive response or acquired resistance, as previously shown in patient-derived xenograft (PDX) models [33,34].

The ability of prostate cells to reversibly alter their lineage identity is responsible for phenotypic plasticity, which is now widely acknowledged as a mechanism of progression in CRPC, driving resistance to next-generation AR signaling inhibitors and leading to the acquisition of neuroendocrine features in approximately 20–25% of cases [35].

Similarly, the study by Sowalsky et al. [36] on radical prostatectomy tissue from 18 men treated with neoadjuvant ADT and abiraterone revealed the phenomenon of lineage plasticity, underlining the presence of *RB1* genomic loss as an early “hit” in the onset of mCRPC. Consequently, in localized primary tumors, we could identify tumors at high-risk of aggressiveness, after which a so comprehensive molecular analysis could help to identify the best therapeutic strategy for eliminating the aggressive subclones in both neoadjuvant and adjuvant settings.

## 4. Biomarker Tools in Prostate Cancer

**Figure 2:**
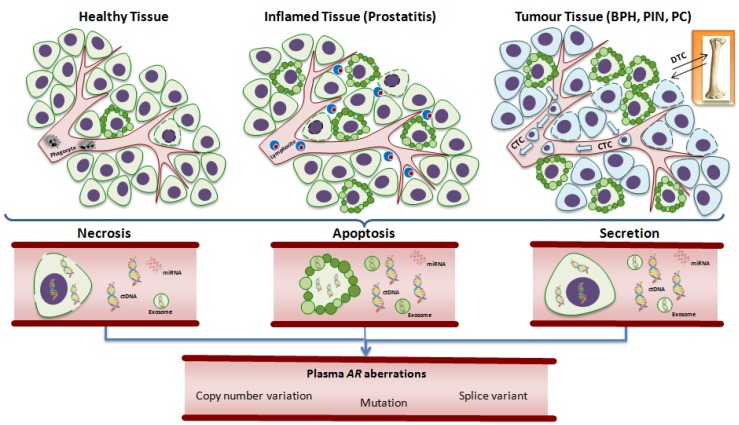
Biology of circulating free DNA. Circulating free DNA (plasma DNA) is released from the cells of healthy, inflamed, or tumor tissue undergoing apoptosis or necrosis, or, more rarely, from living cancer cells that actively release DNA into the circulation because of oncogenic properties. Certainly, levels of plasma DNA are also under the control of the activity of infiltrating phagocytes that usually clear apoptotic and necrotic debris and pro-apoptotic cytokines released by inflammatory cells (e.g., lymphocytes) or cancer cells. Circulating DNA may also be released by CTCs shed by the tumor and by DTC into bone marrow. The analysis of plasma DNA, together with other circulating nucleic acids in the bloodstream, such as miRNA and exosomes, has led to the identification of several genetic and epigenetic alterations of tumor, including circulating aberrations of androgen receptor such as copy number variation, mutations and splice variants. *Abbreviations:* AR, androgen receptor; BPH, benign prostatic hyperplasia; ctDNA, circulating tumor DNA; CTC, circulating tumor cell; DTC, disseminated tumor cell; miRNA, microRNA; PC, prostate cancer; PIN: prostatic intraepithelial neoplasia.

Recent genomic studies have identified distinct molecular subclasses, providing a valuable insight into inter- and intra-patient heterogeneity in different settings of prostate cancer [37,38,39,40,41,42] and showing that genetic changes associated with aggressive disease, when present in early tumors, herald the onset of early biochemical relapse, castration resistance or distant disease progression. However, the majority of these studies evaluated the entire prostate gland to obtain a comprehensive molecular landscape, but this is not always feasible as prostate cancer is often multifocal [43,44] and sometimes also genomically heterogeneous [45], especially in the later stages of the disease, e.g., castration-resistant tumors. Several whole-exome and transcriptome sequencing studies of metastatic tumor biopsies obtained at a single time-point in this setting provided an interesting insight into the complexity and distribution of genomic aberrations [40,46,47] and, given that the procurement of multiple, sequential tumor biopsies from prostate cancer patients is challenging, defining a comprehensive assessment of the changes occurring over time requires more innovative approaches. Consequently, the introduction of liquid biopsies such as circulating tumor cells (CTCs) and circulating tumor DNA (ctDNA) has led to a better monitoring of tumor clone dynamics and a more detailed study of molecular alterations involved in the malignant evolution of prostate cancer (Figure 2).

CTCs in the peripheral blood originate from the primary or metastatic tumor and are involved in the formation of metastasis [48]. Several randomized clinical trials have shown that the enumeration of CTCs is a strong predictive and prognostic biomarker before starting therapy [49,50,51,52] and during treatment [53,54]. Consequently, PCWG3 guidelines [4] recommended the use of CTC enumeration.

Molecular alterations in androgen receptor (AR) on CTCs have been investigated in CTCs to assess their potential as predictors of resistance to treatment. Antonarakis et al. reported an association between AR splice variant-7 (AR-V7) detected in CTCs and resistance to AR-directed therapies in patients with mCRPC [55], but no significant correlation with taxane therapy [56,57]. Recently, a real-time CTC-based assay of nuclear AR expression in the CTCs of CRPC patients was developed using CellSearch System [58]. In addition, phenotypic heterogeneity of CTCs has been shown to inform clinical decision-making between AR-signaling inhibitors and taxanes in mCRPC.

Additional molecular tools evaluating treatment response are also being developed, including the analysis of ctDNA [59,60,61,62,63,64,65,66].

Circulating cell-free DNA (cfDNA) has been detected in the plasma of healthy subjects and men with benign [inflammation, benign prostatic hyperplasia (BPH)] or malignant prostate diseases. Nevertheless, ctDNA, which represents less than 3.0% of total cfDNA, is about three to four times higher in cancer patients than in healthy individuals [67] (Figure 2).

Plasma ctDNA analysis is particularly useful for detecting the evolving therapeutic landscape of mCRPC and for monitoring unpredictable cross-resistance phenomena among agents targeting different pathways such as AR signaling (e.g., enzalutamide and abiraterone) [21,22,68,69], taxane-based chemotherapies [70,71], DNA repair mechanisms [e.g., poly (ADP-ribose) polymerase (PARP) inhibitors] in patients with defects in homologous recombination genes [72], and PI3K/AKT pathway inhibition in *PTEN*-deleted patients [73]. Consequently, there is an urgent need for real-time and practical tumor biomarkers to guide treatment selection and, for this purpose, ctDNA profiling, theoretically available for any patient and at different time-points, could provide a complete overview of genomic information.

The estimation of ctDNA fraction has also been acknowledged as a predictor of prognosis and treatment resistance in mCRPC, whereas there is only preliminary evidence of this in HNPC. Recently, Chi et al. did not observe an increase in the acquisition of AR aberrations (ARV-7, AR amplification, AR ligand binding domain mutations) from cell-free DNA and cfRNA using next-generation sequencing and PCR, respectively, in mHNPC patients treated with apalutamide plus ADT [74].

Moreover, ctDNA tumor fraction may be underestimated in some cases because of the emergence of lesions not included in the targeted gene custom panel used for estimating tumor content.

Despite the fact that ctDNA fraction can also be considered as a useful prognostic biomarker in castration-resistant disease, it should be borne in mind that plasma DNA sequencing is characterized by several limitations involving costs, genome coverage and sensitivity to low ctDNA fraction. Nevertheless, the main limitation of these recent studies on cfDNA concentration remains its inability to specifically quantitate tumor-derived cfDNA fraction in plasma DNA.

However, in general, leveraging plasma specimens collected from different stages of prostate cancer may improve the characterization of key targets for prostate cancer, such as AR, in the real-time and genomic sub-classification of CRPC.

## 5. Detection of Androgen Receptor in Plasma

The persistent activation of the AR pathway, including AR gene amplification and mutations, and the generation of truncated splice variants lacking the ligand-binding domain (LBD) [75], represent a fundamental mechanism of the emergence of castration resistance. The detection of molecular aberrations in CRPC metastatic sites [40,76] was performed with the difficulty of obtaining tissue material amenable to genomic studies and patient consent, which is handled differently in different countries. In prostate cancer, plasma DNA analysis is being increasingly used to identify molecular aberrations but it is not yet a validated test in clinical practice such as and testing of plasma epidermal growth factor receptor (EGFR) mutations, which represents one of current molecular tools in oncology approved by the Food and Drug Administration (FDA) for the treatment selection of EGFR tyrosine kinase inhibitors in mutant lung cancer patients [77]. Recently, the presence of AR amplification and point somatic mutations detected in plasma has been also investigated and associated with a worse outcome in CRPC patients treated with hormonal therapies [59,60,61,62,63,64,65,66].

Plasma *AR* aberrations have been studied using targeted-next generation sequencing (NGS). In several studies a customized AmpliSeq targeted gene panel including *AR* and other gene targets was sequenced on an Ion Torrent Personal Genome Machine or Proton [59,64]. Subsequently, a computational analysis assessed ctDNA fraction, *AR* copy number variation and point mutation detection (with a sensitivity of 98–99%). Recently, our group identified a multiplex digital droplet PCR (ddPCR) to assess *AR* copy number and mutations in plasma cfDNA [64]. Different reference genes (NSUN3, ElF2C1, and AP3B1), and ZXDB at Xp11.21 as a control gene were used to estimate copy number of X chromosome. Each AR CN estimation per reference gene was within two standard deviations from the average AR CN, considering all reference genes and with a high correlation among the individual reference genes. AR mutation detection assays were performed for the AR mutations 2105T > A (p.L702H), 2632A > G (p.T878A) and 2629T > C (p.F877L), with a limit of detection of 1–2% using an input of 2–4 ng of DNA. Conteduca et al. observed a correlation between NGS and ddPCR results for AR copy number and mutations, reporting a strong agreement in both cases (Limits of agreement: mean difference −0.02, 95% confidence intervals (CI) −2.45 to 2.41, and mean difference *p* < 0.001, 95% CI, −0.015 to 0.016, respectively) [64].

Other more common methods available for detecting ctDNA and analyzing plasma *AR* status are summarized in Table 1.

## 6. Plasma Androgen Receptor (AR) and Hormonal Treatments

AR gene aberrations have been shown to be uncommon in the early stages of prostate cancer, but very frequent in CRPC. In fact, NGS- and PCR-based studies have focused in particular on *AR* copy number in advanced disease, mainly in a post-docetaxel setting. Romanel et al. [59] sequenced 274 plasma samples from 97 CRPC patients treated with abiraterone, reporting that plasma *AR* gain and point mutations were mutually exclusive, and that *AR* copy number was unchanged during abiraterone treatment. Moreover, the onset of T878A or L702H AR amino acid changes was reported in 13% of samples at progression on abiraterone. The association between plasma *AR* aberrations and outcome was very interesting, revealing a letter reduction in PSA in AR-aberrant patients than in AR-normal patients, and significantly shorter OS [hazard ratio (HR) 7.33, 95% CI 3.51–15.34, *p* = 1.3 × 10^−9^) and progression-free survival (PFS) (HR 3.73, 95% CI 2.17–6.41, *p* = 5.6 × 10^−7^). Subsequently, Conteduca et al. assessed the role of plasma *AR* not only in a post-docetaxel group, but also in a chemotherapy-naïve setting [64]. A primary cohort including 73 chemotherapy-naïve and 98 post-docetaxel patients treated with enzalutamide or abiraterone were evaluated between January 2011 and June 2016 and independently recruited to biomarker protocols at the Royal Marsden (London, UK) or IRST IRCCS (Meldola, Italy). Ten (14%) pre-chemotherapy and 33 (34%) post-docetaxel showed *AR* gain. Eight (11%) post-docetaxel but no chemotherapy-naïve abiraterone-treated patients were *AR* mutant. Chemotherapy-naïve and post-docetaxel *AR*-gained patients had a worse outcome (OS: HR = 3.98, 95% CI 1.74–9.10, *p* < 0.001 and HR = 3.81, 95% CI 2.28–6.37, *p* < 0.001, respectively, and PFS: HR = 2.18, 95% CI 1.08–4.39, *p* = 0.03, and HR = 1.95, 95% CI 1.23–3.11, *p* = 0.01, respectively). Patients with *AR* mutations also had a significantly shorter OS (HR = 3.26, 95% CI 1.47–not reached, *p* = 0.004). These data were validated in a secondary cohort of 94 chemotherapy-naïve patients treated with enzalutamide in 16 institutions in the PREMIERE trial (NCT02288936). Eleven (12%) had *AR* gain. AR-gained patients had worse biochemical PFS (HR = 4.33, 95% CI 1.94–9.68, *p* < 0.001), radiographic PFS (HR = 8.06, 95% CI 3.26–19.93, *p* < 0.001), OS (HR = 11.08, 95% CI 2.16–56.95, *p* = 0.004). Subsequently, plasma *AR* was associated with different prognostic/predictive factors to identify additional mechanisms of resistance to AR-directed therapies.

After establishing the prognostic/predictive role of plasma *AR* gain in CRPC patients receiving AR signaling inhibitors, Conteduca et al. evaluated its association with other biomarkers, such as choline uptake in 18F-fluorocholine positron emission tomography/computed tomography (FCH–PET/CT) [78,79]. Eighty post-docetaxel CRPC patients treated with abiraterone (n = 47) or enzalutamide (n = 33) were evaluated and, a robust correlation was observed between *AR* gain and some FCH–PET/CT parameters (tumor lesion activity and metabolic tumor volume), both of which can be considered as independent factors of PFS and OS in multivariate analysis.

An additional association was observed between plasma AR and serum concentration of chromogranin A (CGA) [80,81], which is considered a marker of neuroendocrine differentiation (NED), an alternative mechanism of resistance to hormonal treatments. Neuroendocrine prostate cancer (NEPC) is a rare, lethal subtype of prostate cancer characterized by AR-independence, reduced PSA level, and usually by the expression of neuroendocrine markers, such as CGA, synaptophysin, and neuron-specific enolase (NSE) [82]. A recent study [83] showed that patients with adenocarcinoma harboring specific clinical and molecular features (e.g., liver metastases, slight increase in PSA, high levels of neuroendocrine markers, TP53 or RB1 alterations) before a confirmed clinical diagnosis of therapy-related NEPC (especially after abiraterone or enzalutamide treatment) were considered to have a high risk of developing NED. Thus, Conteduca et al. assessed the impact of combining serum CGA level and plasma *AR* copy number by ddPCR on the outcome of 256 CRPC patients treated with AR-directed therapies [84]. Plasma *AR* gain and high pre-treatment levels of serum and LDH were viewed as independent predictors of PFS and OS, suggesting the utility of assessing serum CGA before starting abiraterone or enzalutamide treatment together with plasma *AR* status to identify a subgroup of CRPC patients who are not likely to respond to AR-directed agents but have no AR aberrations.

## 7. Plasma Androgen Receptor (AR) and Chemotherapy

Docetaxel and cabazitaxel represent the most widely used chemotherapies for patients with mCRPC. They have a similar function, preventing mitosis and microtubule-dependent trafficking and eliciting apoptosis. In addition, they may block nuclear translocation and AR activity [85]. The role of plasma *AR* gain in patients treated with taxanes was explored in two multi-institutional studies [86,87] that performed a pooled analysis of AR status, determined by droplet digital PCR, on pre-treatment plasma samples. The first biomarker study evaluated the association between plasma *AR* and OS/PFS and PSA response rate in 163 docetaxel-treated patients, reporting only a significant shorter OS in *AR*-gained patients (HR = 1.61, 95% CI 1.08–2.39, *p* = 0.018). In addition, the same authors studied the interaction between plasma *AR* and treatment type in 73 patients of the abiraterone/enzalutamide-treated group (after incorporating updated data from their prior study [64]) and 115 first-line docetaxel patients. In first-line therapy group, findings suggested that *AR*-normal patients could benefit from hormonal treatment, while plasma *AR*-gained subjects had a better response to docetaxel. Similar results were observed for second-line therapy mCRPC patients, with a meaningful treatment interaction between plasma *AR* and cabazitaxel vs. AR-directed therapies for OS (*p* = 0.041). In addition, Conteduca et al. performed an exploratory analysis of an AR-gained cabaxitaxel cohort, revealing that these patients, treated with an initially reduced dose of cabazitaxel, had a significantly shorter OS/PFS and would probably need a standard initial dose of cabazitaxel. Plasma *AR* status was shown to have a potential clinical utility in patients being considered for treatment with taxanes.

## 8. Plasma Androgen Receptor (AR) and Novel Drugs

In a phase 2 clinical trial (NCT03454750) of mCRPC patients treated with ^177^Lu–PSMA–617, plasma *AR* gene status was also assessed in association with clinical outcome [88]. Early progressive disease was reported in 17 (42.5%) of the 40 patients (12 of 15 (80%) with *AR* gene gain and 5 of 25 (20%) with *AR* normal (*p* = 0.0002)). The OR for patients with early disease progression and *AR* gain was 16.00, 95% CI 3.23–79.27, *p* = 0.0007. These preliminary data suggest that plasma *AR* status determined by ddPCR could be useful to identify mCRPC patients resistant to ^177^Lu–PSMA–617.

Recently, preclinical data indicated a synergy between olaparib and androgen pathway inhibitors. Specifically, a randomized, double-blind, placebo-controlled, phase 2 trial assessed the efficacy of olaparib plus the androgen pathway inhibitor abiraterone in patients with mCRPC regardless of HRR mutation status, results showing the clinical benefit of the combination compared with abiraterone alone [89]. Plasma *AR* status could thus help to increase clinical benefit in a broader population of mCRPC.

In addition, recent studies assessed the role of AR mutations, e.g., F877L and T878A, in the resistance to next-generation AR-directed therapies, such as apalutamide [90] and darolutamide [91]. The phase I/II study ARN-509-001 [90] used the sensitive BEAMing assay to detect AR mutations in plasma DNA in apalutamide-treated nonmetastatic CRPC and mCRPC patients. However, the overall frequency of *AR* mutations was very low, underlining that they could be not considered as a helpful biomarker to identify acquired resistance to apalutamide.

Recent preclinical evidence [92] has also emerged of the activity of darolutamide in enzalutamide-treated CRPC, revealing the activity of darolutamide to significantly inhibit the transcriptional action of F877L, H875Y/T878A, F877L/T878A, and T878G AR mutants, which transform enzalutamide into a partial agonist. These preliminary preclinical results could be corroborated in ctDNA assays are capable of detecting AR mutants sensitive to darolutamide or other novel hormonal drugs, in a precision oncology setting.

## 9. Conclusions

The current therapeutic and molecular landscape of prostate cancer is rapidly changing and has led to novel findings not only in CRPC, but also in earlier stages of the disease. In this context, the identification of predictive and prognostic biomarkers is essential, and it is possible thanks to the use of plasma DNA to monitor tumor dynamics and treatment outcome. In prostate cancer, plasma *AR* status, given its role in patients treated with different drugs, could become an important predictive biomarker to guide treatment selection in mCRPC, along with other factors such as PSA level. Indeed, plasma *AR* status has also recently been assessed in the context of a novel potential prognostic index model for survival including clinical, imaging, and molecular factors [93].

In conclusion, the integration of different biomarker strategies, including genomics, with plasma *AR* status in prostate cancer, could substantially improve prognostication and stratification of these patients. A prospective evaluation of plasma *AR* as a biomarker of treatment selection is warranted with a possible validation cohort, and there is an urgent need to assess the utility of plasma *AR* not only in advanced patients, but also in those with earlier stages of the disease.

## Figures and Tables

**Table 1:** Most common methods used to detect circulating tumor DNA (ctDNA) and analyse plasma androgen receptor (AR) status.

Method	Advantages	Limitations
**Focused**		
Quantitative real time PCR	Variable sensitivity with detection limit <1% (0.01% for digital PCR, PAP-A and BEAMing) Easy and rapid to use. Less expensive. Multiplex ddPCR can simultaneous screening for multiple mutations from the same sample	Necessary known hotspots in selected genes (or single probes for rare variants designed on a ‘personalized’ basis).
Fluorescence- labeled PCR		
Nested real time PCR		
ARMS-Scorpion PCR		
PAP-A		
BEAMing		
ddPCR		
Microfluidic digital PCR		
Mass spectrometry		
**Targeted**		
PARE	High depth and sensitivity of analysis with detection limit 2% (0.1% and 0.01% for Tam-Seq and CAPP-Seq, respectively). De novo mutation identification. More comprehensive analysis across wider genomic regions	Very costly Requirement for high-quality DNA Extensive data analysis requiring a dedicated bioinformatician
Tam-Seq		
Safe-Seq		
CAPP-Seq		
Ion-Ampliseq		
**Broad**		
WES/WGS	High sensitivity with detection limits 1–5%. Characterization a large spectrum of the genome, without the need to focus on predefined or existing alterations. More in-depth interrogation of multiple regions (WES > WGS)	Very costly Requirement for high-quality DNA Extensive data analysis requiring a dedicated bioinformatician. To accurately detect clinical mutations, a 100-to 200-fold sequencing coverage (number of times the genome is sequenced) may be needed, which are both time and cost prohibitive. Low sensitivity for the identification of copy number variation (WES)

Abbreviations: ARMS, amplification refractory mutation system; BEAMing, beads, emulsion, amplification, magnetics; CAPP-Seq, cancer personalized profiling by deep sequencing; HCC, hepatocellular cancer; NSCLC, non-small cell lung cancer; PAP–A, pyrophosphorolysis-activated polymerization-allele-specific amplification; PARE, personalized analysis of rearranged ends; Safe- SeqS, safe-sequencing system; TAm–Seq, tagged amplicon deep sequencing; WGS, whole genome sequencing; WES, whole exome sequencing.
